# Comparing women pharmacy consumers’ experiences with weight loss treatment in Victoria and Nottingham: a cross-sectional study

**DOI:** 10.1186/1471-2458-14-662

**Published:** 2014-06-28

**Authors:** Souhiela Fakih, Jennifer L Marriott, Helen Boardman, Claire Anderson, Safeera Y Hussainy

**Affiliations:** 1 Centre for Medicine Use and Safety, Monash University, Parkville, Australia; 2 Division of Social Research in Medicines and Health School of Pharmacy, University of Nottingham, Nottingham NG7 2RD, UK

**Keywords:** Women, Body weight, Weight loss, Health services, Community pharmacy services, Health knowledge, attitudes, practice

## Abstract

**Background:**

There has been a recent increase in weight management services available in pharmacies across Australia and England. The aim of this study was to determine the following between women in Victoria and Nottingham: similarities and differences of what weight management options are preferred by women pharmacy consumers; how they feel about pharmacists providing advice in this area; and what they desire in a weight management program.

**Method:**

Women pharmacy consumers were randomly approached by a researcher in community pharmacies in Victoria and Nottingham and asked to complete a questionnaire regarding their own weight management experiences. The questionnaire was self-completed or researcher-administered and was comprised of four main sections that focused on the participant’s general health, previous weight loss experiences, their ideal weight management program and their demographics. Data was entered in SPSS 19 and logistic regression was used to identify any differences in weight loss experiences between women.

**Results:**

The participant rates were high: 86% (n = 395/460) in Victoria and 98% in Nottingham (n = 215/220). Overall, women in Victoria and Nottingham were similar with comparable demographics. Approximately 50% (250/507) of women were in the overweight or obese body mass index category, with over 70% (n = 436/610) of women having attempted to lose weight in the past. The majority of women (n = 334/436) felt comfortable receiving advice from pharmacists. In the logistic regression analysis women in Nottingham were found to be significantly less likely to have utilised a pharmacy weight management program in the last five years (OR: 0.23 CI: 0.08, 0.63) and were significantly less likely to want an ideal weight management program located in a pharmacy (OR: 0.49 CI: 0.30, 0.82) compared to women in Victoria. No significant associations between location and feeling comfortable with a pharmacist advising on weight loss or wanting a pharmacist in an ideal weight management program were seen.

**Conclusion:**

Results from this study have provided information on possible ideal pharmacy weight management programs in both Victoria and Nottingham. Although differences were seen between the two populations, similarities between ideal weight management programs and comfort level with pharmacist interaction were noted.

## Background

According to the WHO one billion adults are overweight and at least 300 million adults are obese worldwide [1]. Australia has one of the highest overweight and obese populations in the world, with over two-thirds of the population being considered overweight or obese [2]. Similarly, in England, an estimated 41% of males and 33% of females are considered overweight and 24% of males and 26% of females are estimated to be obese [3]. With the rising prevalence of overweight and obesity in both Australia and England, key stakeholders and government bodies have communicated the urgency of developing public health interventions to target this epidemic [4,5].

In both Australia and England, a plethora of products and programs are marketed for weight loss, with many now offered in community pharmacies. However, even with the increase in weight management options available, combination approaches of diet, exercise and behavioural modification are still considered first line [6-8]. Many of the over-the-counter (OTC) products, such as herbal supplements, which are marketed for weight loss, lack evidence and are not recommended in weight management guidelines [6,8].

Women are said to be the major purchasers of weight loss products and programs, and reportedly try to lose weight more frequently than their male counterparts [9]. There are specific causes of overweight and obesity specifically related to women, including polycystic ovary disease, hormonal changes, pregnancy and menopause [10]. In women, overweight and obesity increases the risk of infertility and pregnancy complications [11]. It is for these reasons that specifically investigating women’s needs, to devise an appropriate weight management intervention for women, has been recommended [12].

Community pharmacists have been increasingly recognised as key health care professionals to help combat the overweight and obesity issue due to their easy accessibility, diverse clientele and high level of patient trust [13-17]. Consumers visit community pharmacies on a regular basis, with reports documenting that the average Australian visits a pharmacy up to 14 times a year [18]. More specifically, in Australia, pharmacy staff (pharmacists and pharmacy assistants) come into contact with women in the pre-pregnancy, pregnancy and post-partum stages more than any other primary health care professional, including general practitioners and nurses [19]. In the UK, consumer access to pharmacies and pharmacists is similar to Australia, with 60-80% of pharmacy consumers reported to be women [20].

Recent studies have reported the need for increased training of pharmacists and pharmacy assistants in the weight management area so that pharmacies can provide an ideal health destination for consumers to receive evidence-based services and advice [19,21,22]. It is well known that before any interventions or education resources are developed, an important component is to involve consumers, draw from their experiences, and take into account their attitudes towards weight management approaches [23]. Previous studies have focussed on the general public’s perceptions of weight management services offered by community pharmacies, with little focus on actual pharmacy consumers [24-26]. To date, Fakih *et al.* are the only research group who have previously explored women pharmacy consumers’ experiences with weight management approaches in Australia [27]. They highlighted women pharmacy consumers’ positive attitudes towards pharmacy involvement in weight management counselling and emphasised the importance of adequately trained health care professionals in weight management.

With England and Australia offering similar pharmacy services, the primary aim of this study was to determine whether women pharmacy consumers in both countries have similar experiences with, and attitudes towards, weight management, specifically:,

• their awareness of potential consequences of obesity;

• their experience using weight loss treatments;

• treatment duration, perceived benefits and adherence;

• level of interaction with health professionals during treatment;

• advice given;

• comfort level with pharmacists providing weight management advice; and

• components of an ideal weight management program.

The secondary aim of this study was to determine whether future weight management interventions, guidelines or educational resources could be developed for use in either country.

## Methods

### Survey development

Women pharmacy consumers were surveyed using a questionnaire comprising four main sections that focused on the participant’s general health, previous weight loss experiences, their ideal weight management program and their demographics. Two questionnaires were developed; one for women in Victoria, Australia to complete and one for women in Nottingham, England. The questionnaires were very similar, with the only differences being the units of measurement used and the names of weight loss products. Only women who had previously attempted to lose weight were required to complete the sections regarding previous weight loss experiences and their ideal weight management program.

The first drafts of the questionnaires were piloted for face and content validity by academics, pharmacists and consumers. Changes regarding formatting and wording of certain questions were made based on their feedback. The questionnaires were then piloted in two pharmacies with women pharmacy consumers (n = 20). Any issues, mostly formatting and space given to open ended questions, were then addressed and the final questionnaires were printed (see Additional files 1 and 2).

### Study participants

As this was an exploratory study, the sample size was determined based on allocated resources. The aim was to collect data from 300 women over a three-month data collection period (January-March 2011) in Victoria and 200 women over a six-week data collection period in Nottingham (February-March 2011).

Women pharmacy consumers were recruited from 34 pharmacies across Victoria and 15 pharmacies around Nottingham. Pharmacies were randomly selected using the Pharmacy Board of Victoria pharmacy premises list or the National Health Service Nottingham pharmacy list. Sixty-five pharmacies from Victoria were contacted and 34 pharmacies agreed to be involved. In Nottingham, recruitment was more difficult; the researcher (S.F.) was unable to administer the questionnaire in large pharmacy chains such as Boots® because of delays in permission by the chain group pharmacies. S.F. could therefore only recruit independently owned community pharmacies in Nottingham. Twenty-four independently owned pharmacies were contacted and 15 agreed to participate. Pharmacies were visited on weekdays and weekends and during different times between 8 am to 9 pm to ensure a representative sample of women pharmacy consumers would be recruited. Each pharmacy was only visited once for 6–8 hours on a mutually convenient date.

### Survey administration

The survey was administered to women pharmacy consumers in Victoria by one of the researchers (S.F.) or a research assistant (J.W.) during January-March 2011. In Nottingham, the survey was administered during February-March 2011 by S.F. Women over the age of 18 who were able to independently complete a questionnaire in English were asked to complete the study questionnaire in the pharmacy. Participants were approached at random and were asked to complete the questionnaire, regardless of whether they were underweight, healthy or overweight.

Participants were given the choice of having the questionnaire filled in by the researcher on their behalf (researcher-administered) or self-completing the questionnaire. Participants were not given the opportunity to complete the questionnaire in any other location or at any other time.

All participants who completed the questionnaire received an AU $7.50 voucher in Victoria or a £5 voucher in Nottingham to spend in the pharmacy on non-prescription products.

### Data analysis

Data were analysed using SPSS version 19.0 (IBM, USA) and summarised using descriptive statistics. Multiple response questions were coded yes or no for each response i.e. multiple-dichotomy method. Pearson’s chi-squared test was used to determine any significant relationships between women in Victoria and Nottingham. The association between pharmacy-specific outcomes, such as wanting a pharmacist involved in an ideal weight management program and demographic characteristics, were investigated using multivariate logistic regression. The significance level was set at P < 0.05.

To explore relationships between different stages in a woman’s life (pre-pregnancy, pregnancy, pregnancy, post-partum and menopause) the age categories for women were collapsed into three main categories: 18–30, 31–50 and over 50. Body mass index (BMI) was calculated using height and weight provided, and categories were selected based on BMI cut-off points provided by the WHO [1].

The study was approved by the Monash University Human Research Ethics Committee and the University Of Nottingham School Of Pharmacy Ethics Committee.

## Results

In total, there were 610 participating women in Victoria and Nottingham. The overall participant rate was 86% in Victoria (395 completed surveys from 460 women approached) and 98% in Nottingham (215 completed surveys from 220 women approached).

### Characteristics of women

The characteristics of the participating women in Victoria and Nottingham were comparable (Table 1). With increasing age women’s BMI also increased. This trend was seen in both Victoria and Nottingham.

**Table 1:** Demographic characteristics of participating women

**Demographic characteristic**	**Percentage of women Victoria**	**Percentage of women Nottingham**	***P-*** **value**
	**n (%); **^**a**^**N = 395**	**n (%); **^**b**^**N = 215**	
**Age (in years)**			
18-30	85 (21.7)	48 (22.4)	0.80
31-50	149 (38.0)	91 (42.5)	0.22
≥51	158 (40.3)	75 (35.0)	0.23
**Education**			
Secondary school or less	155 (39.4)	100 (46.5)	0.08
Post-secondary school certificate	78 (19.8)	56 (26.8)	0.05
University student/graduate	124 (31.6)	36 (16.7)	*<0.001*
Post-graduate	36 (9.2)	17 (8.1)	0.67
**BMI kg/m**^2^**	*N = 319*	*N = 188*	
Underweight *<18.5*	9 (2.8)	6 (3.2)	0.82
Healthy *18.5-24.9*	157 (49.2)	85 (45.2)	0.36
Overweight *25.0-29.9*	77 (24.1)	57 (30.3)	0.11
Obese *30.0-39.9*	68 (21.3)	38 (20.2)	0.75
Severely obese *>40*	8 (2.5)	2 (1.1)	0.26
**Smoking status**			
Current	71 (18.0)	53 (24.8)	*0.05*
Never	273 (69.3)	100 (46.7)	*<0.001*
Former	50 (12.7)	61 (28.5)	*<0.001*
**Medical conditions**			
Yes	212 (54.2)	126 (58.9)	0.27
**Medications**			
Yes	271 (68.8)	144 (68.6)	0.96
**Health care professional(s) (HCP) visited in the last 12 months**			
Doctor	369 (93.7)	189 (88.3)	*0.02*
Pharmacist	296 (75.1)	106 (49.5)	*<0.001*
Dentist	219 (55.6)	134 (62.6)	0.09
Optometrist	138 (35.0)	64 (29.9)	0.20
Dietitian	29 (7.4)	4 (18.7)	*0.004*
Physiotherapist	69 (17.5)	15 (7.0)	*<0.001*
^c^Other	163 (41.4)	42 (19.6)	*<0.001*
**Most visited HCP in the last 12 months**			
Doctor	226 (58.1)	125 (59.2)	0.786
Pharmacist	78 (20.1)	44 (20.9)	0.816
^d^Other	83 (21.3)	42 (19.9)	0.680
**Previously attempted to lose weight**			
Yes	281 (71.1)	155 (72.1)	0.803

^a^N = 395; totals do not total 395 due to missing responses, ^b^N = 215; totals do not total 215 due to missing responses.

**BMI could only be calculated for the women who self-reported both their height and weight (Approximately 20% (77/395) of women in Victoria and 12.5% (27/215) of women in Nottingham did not provide enough information for their BMI to be calculated.).

^c^Other = podiatrist, psychologist and “other” responses.

^d^Other = all other health care professionals including, dentist, psychologist, physiotherapist, dietitian, optometrist and podiatrist.

### Similarities and differences between women in Victoria and Nottingham: weight loss perceptions, methods used, experiences with treatments and advice received from health care professionals (HCPs)

Women in Victoria were significantly more likely (P = 0.005) to utilise increased exercise and decreased calorie intake as part of their weight loss method compared to women in Nottingham who were more likely to be involved in a weight loss support group (P < 0.001) (Table 2). Women in Victoria were also significantly more likely to utilise pharmacy based programs compared to women in Nottingham (P = 0.006).

**Table 2:** Comparison between weight loss experiences in women from Victoria, Australia and women in Nottingham, England

**Weight loss perceptions, practices and interactions with health care professionals**	**Victoria**	**Nottingham**	***P-*** **value**
	^**a**^**N = 281; n(%)**	^**b**^**N = 155; n(%)**	
**Why did you want to lose weight?**			
To look and feel good	162 (58.1)	112 (73.7)	*0.001*
For a special event	58 (20.8)	24 (15.8)	0.21
For my health	196 (70.3)	95 (62.5)	0.10
Someone told me to	12 (4.3)	6 (3.9)	0.86
Other e.g. wanted to fall pregnant	21 (7.5)	6 (3.9)	0.14
**Weight loss methods used in the last five years**			
Decreased calorie intake	240 (86.3)	126 (81.8)	0.21
Increased exercise	236 (84.9)	105 (68.6)	*<0.001*
Decreased calorie intake and increased exercise	212 (76.3)	96 (62.7)	*0.005*
Weight loss support group e.g. Jenny Craig® or Weight Watchers®	57 (20.5)	57 (37.0)	*<0.001*
Decreased calorie intake, increased exercise and weight loss support group	44 (15.8)	34 (22.2)	*0.022*
Meal replacement products e.g. Optifast®	35 (12.6)	24 (15.6)	0.39
Pharmacy based program e.g. Tony Ferguson®	33 (11.9)	6 (3.9)	*0.006*
Weight loss medication e.g. Xenical®	11 (4.0)	8 (5.2)	0.55
Vitamins/herbal products	29 (10.4)	9 (5.8)	0.11
Other e.g. recreational drugs, weight loss surgery	14 (5.0)	5 (3.2)	0.39
**Time since last weight loss attempt (months)**			
Median (IQR)	12 (6–34)	12 (6–25.5)	0.83
**Last place purchased weight loss method**			
Not applicable	195 (73.3)	102 (69.4)	0.40
Supermarket	8 (3.0)	16 (10.9)	*0.001*
Pharmacy	49 (18.4)	14 (9.5)	*0.02*
Other e.g. internet, health food store	14 (5.3)	15 (10.2)	0.06
**How much weight was wanting to be lost**			
0-2 kgs	17 (6.4)	10 (6.5)	0.97
3-5 kgs	81 (30.7)	28 (18.3)	*0.006*
6-10 kgs	95 (36.0)	45 (29.4)	0.17
11-15 kgs	24 (9.1)	26 (17.0)	*0.02*
16-20 kgs	14 (5.3)	18 (11.8)	*0.01*
Over 20 kgs	33 (12.5)	26 (17.0)	0.20
**How much weight was lost**			
0-2 kgs	68 (26.2)	31 (20.7)	0.21
3-5 kgs	97 (37.3)	35 (23.3)	*0.004*
6-10 kgs	59 (22.7)	38 (25.3)	0.54
11-15 kgs	13 (5.0)	23 (15.3)	*<0.001*
16-20 kgs	11 (4.2)	7 (4.7)	0.84
Over 20 kgs	12 (4.6)	18 (10.7)	*0.006*
**Duration of weight loss attempt**			
0-3 weeks	21 (8.2)	16 (10.5)	0.44
1-2 months	80 (31.3)	35 (22.9)	*0.07*
3-5 months	68 (26.6)	26 (17.0)	*0.03*
6-8 months	39 (15.2)	30 (19.6)	0.25
9-11 months	15 (5.9)	19 (12.4)	*0.02*
Over 1 year	33 (12.9)	27 (17.6)	0.19
**Has the weight lost since been regained**			
Yes	147 (57.2)	80 (53.3)	0.45
**Health care professional advice during last weight loss attempt**			
No advice	182 (67.4)	126 (82.4)	*0.001*
Doctor	45 (16.7)	9 (5.9)	*0.001*
Exercise consultant	19 (7.0)	9 (5.9)	0.65
Dietitian	7 (2.6)	2 (1.3)	0.38
Pharmacist	23 (8.5)	5 (3.3)	*0.04*
Pharmacy assistant	6 (2.2)	4 (2.6)	0.80
Other e.g. Weight Watchers® consultant	15 (5.6)	5 (3.3)	0.29
**Most trusted source for weight management advice**			
Family/friends	86 (32.3)	39 (26.2)	0.19
Internet	40 (15.0)	10 (6.8)	*0.01*
TV/radio	10 (3.8)	5 (3.4)	0.84
Exercise consultant	30 (11.3)	9 (6.1)	*0.08*
Health care professional	143 (53.8)	24 (16.2)	*<0.001*
No one	33 (12.4)	52 (35.1)	*<0.001*
**Biggest problems in weight management**			
No problems	15 (5.5)	19 (12.3)	*0.01*
Lack of motivation	207 (75.3)	96 (61.9)	*0.004*
Lack of support from family/friends	14 (5.1)	12 (7.7)	0.27
Lack of time	169 (61.5)	70 (45.2)	*0.001*
Lack of support from HCP	1 (0.4)	3 (1.9)	0.10
Cost of product or program	101 (36.7)	28 (18.1)	*<0.001*
Currently available products don’t work	5 (1.8)	4 (2.6)	0.60
Not enough information	10 (3.6)	3 (1.9)	0.32
Side effects	6 (2.2)	7 (4.5)	0.18

^a^N = 281; totals do not total 281 due to missing responses, ^b^N = 155; totals do not total 155 due to missing responses.

Women in Victoria were significantly less likely to want to lose more than 10kgs in their last weight loss attempt compared to women in Nottingham (P < 0.001). Women in Victoria were also less likely to have lost more than 10kgs in their last weight loss attempt compared to women in Nottingham (P < 0.001). Furthermore, women in Victoria were less likely to state that they had used their last weight loss method for six months and over compared to women in Nottingham (P = 0.002).

**Figure 1:**
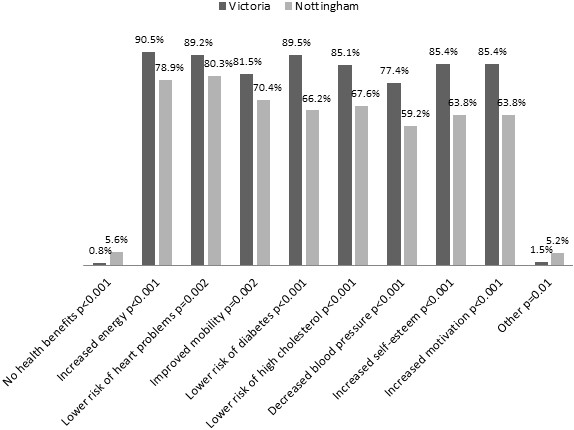
Perceived benefits of weight loss by women.

More women in Victoria (90/272) received advice from a health care professional in their last weight loss attempt compared to women in Nottingham (27/153). Advice received from health care professionals was in line with national guidelines for both women in Victoria and women in Nottingham, with approximately 80% of health care professionals recommending diet and exercise (Victoria: 77.8%, (70/90); Nottingham: 81.5%, (22/27)). The majority of women in both Victoria (93.3%; 84/90) and Nottingham (88.9%; 24/27) who received advice from a health care professional also found the advice helpful. Both groups felt somewhat comfortable or extremely comfortable with receiving advice on weight management from their community pharmacist (Victoria 82.2%, (231/281); Nottingham, 74.2% (115/155)).Overall, women in Victoria were significantly more likely to recognise the health benefits of losing weight compared to women in Nottingham (Figure 1). In both groups, the least recognised benefit was decreased blood pressure.

### Differences and similarities between pharmacy specific outcomes

Women who had sought a pharmacist’s advice on health, in the last 12 months, were significantly more likely to want a pharmacist in their ideal weight management program (OR: 2.29 CI: 1.35, 3.90) and preferred their ideal weight management program to be located in a pharmacy (OR: 3.11 CI: 1.75, 5.53) compared to women who had not sought advice from a pharmacist (Table 3). Women in Nottingham were significantly less likely to have utilised a pharmacy weight management program in the last five years (OR: 0.23 CI: 0.08, 0.63) and were significantly less likely to want an ideal weight management program located in a pharmacy (OR: 0.49 CI: 0.30, 0.82) compared to women in Victoria. No other significant associations between demographic characteristics and utilising a pharmacy-based program, feeling comfortable with a pharmacist advising on weight loss, wanting a pharmacist in an ideal weight management program or having an ideal weight management program located in a pharmacy were found (Table 3).

**Table 3:** **Adjusted odds ratio (95**% CI)** for associations between demographic characteristics and various pharmacy specific outcomes (N = 436)

	**Utilised pharmacy weight management programs in the last five years**	**Feeling comfortable**^**a **^**with pharmacists giving advice on weight loss**	**Wanting a pharmacist in an ideal weight management program**	**Wanting an ideal weight management program to be located in a pharmacy**
	**(yes; n = 39)**	**(yes; n = 334)**	**(yes; n = 171)**	**(yes; n = 157)**
	**OR (95% CI)**	**OR (95% CI)**	**OR (95% CI)**	**OR (95% CI)**
**Location survey was administered**				
Victoria	1.0	1.0	1.0	1.0
Nottingham	0.23 (0.08, 0.63)	0.67 (0.39, 1.16)	0.71 (0.44, 1.12)	0.49 (0.30, 0.82)
**Age**				
18-30	1.0	1.0	1.0	1.0
31-50	1.12 (0.42, 3.0)	0.56 (0.26, 1.23)	0.45 (0.26, 0.88)	0.85 (0.45, 1.62)
≥50	0.72 (0.24, 2.13)	0.60 (0.26, 1.38)	0.83 (0.43, 1.58)	0.84 (0.42, 1.65)
**Medical conditions**				
No	1.0	1.0	1.0	1.0
Yes	1.36 (0.51, 3.61)	1.18 (0.59, 2.34)	0.93 (0.52, 1.68)	0.61 (0.32, 1.14)
**Taking medications**				
No	1.0	1.0	1.0	1.0
Yes	0.85 (0.37, 1.96)	0.98 (0.42, 2.01)	0.77 (0.42, 1.45)	1.11 (0.57, 2.16)
**Visited pharmacist in the last 12 months**				
No	1.0	1.0	1.0	1.0
Yes	0.85 (0.37, 1.96)	1.57 (0.88, 2.80)	2.29 (1.35, 3.90)	3.11 (1.75, 5.53)
**BMI**^**c**^				
<25 kg/m^2^	1.0	1.0	1.0	1.0
≥25 kg/m^2^	1.70 (0.76, 3.79)	1.0 (0.57, 1.77)	1.13 (0.70, 1.82)	1.1 (0.66, 1.81)

Note: OR = odds ratio; CI = confidence interval.

**Adjusted for location, age, medical conditions, medications, if visited pharmacist in the last 12 months and BMI.

^a^Feeling comfortable: comfort level categories have been collapsed into two categories; feeling comfortable (participants who were somewhat comfortable or extremely comfortable) and not feeling comfortable (participants who were, unsure, not comfortable and extremely not comfortable).

^c^BMI: BMI categories have been collapsed into two categories; <25 mg/kg^2^ and ≥25 mg/kg^2^.

### Ideal weight management program

Overall, women’s views on the features of an ideal weight management program results were comparable amongst the women in Victoria and Nottingham (Table 4). Women in both Victoria and Nottingham and across all age groups wanted information on weight management to be delivered face-to-face. Women in Victoria were more likely to want a health care professional involved in their ideal weight management program compared to women in Nottingham (P = 0.008). Women in Nottingham were more likely to want their program to be located in their workplace than in a pharmacy (P = 0.04). Differences in an ideal weight management program were seen between age groups. This pattern was the same for women in both Victoria and Nottingham. Table 4 highlights women pharmacy consumers’ ideal weight management program in Victoria and Nottingham and the differences seen amongst women in varying age groups.

**Table 4:** Women pharmacy consumers’ ideal weight management program in Victoria and Nottingham according to different age-groups

**Ideal feature**	**Victoria**	**Nottingham**	***P-*** **value**
	^**a**^**N = 281; n(%)**	^**b**^**N = 155; n(%)**	
**Information delivery**			
***Face-to-face***	215 (79.9)	117 (76.0)	0.34
18-30	52 (83.9)	25 (75.8)	0.34
31-50	76 (73.8)	52 (77.6)	0.57
≥51	86 (83.5)	40 (75.5)	0.23
***Email***	76 (28.3)	41 (26.6)	0.72
18-30	25 (40.3)	12 (36.4)	0.71
31-50	42 (40.8)	23 (34.3)	0.40
≥51	9 (8.7)	6 (11.3)	0.60
***Telephone call***	9 (3.3)	11 (7.1)	0.77
18-30	4 (6.5)	3 (9.1)	0.64
31-50	2 (1.9)	5 (7.5)	0.08
≥51	3 (2.9)	3 (5.7)	0.40
***Mobile phone***	11 (4.1)	11 (7.1)	0.17
18-30	8 (12.9)	7 (21.2)	0.29
31-50	3 (2.9)	4 (6.0)	0.33
≥51	0	0	1
***Postal letter***	16 (5.9)	17 (11.0)	0.06
18-30	0	5 (15.2)	*0.002*
31-50	9 (8.7)	7 (10.4)	0.71
≥51	7 (6.8)	4 (7.5)	0.86
**HCPs needed**			
***None***	20 (7.4)	24 (15.6)	*0.008*
18-30	5 (8.2)	2 (6.1)	0.71
31-50	7 (6.7)	15 (22.4)	*0.003*
≥51	8 (7.8)	6 (11.3)	0.46
***Doctor***	178 (65.9)	50 (32.5)	*<0.001*
18-30	38 (62.3)	9 (27.3)	*0.001*
31-50	64 (61.5)	20 (29.9)	*<0.001*
≥51	76 (73.8)	21 (39.6)	*<0.001*
***Dietitian***	188 (69.6)	74 (48.1)	*<0.001*
18-30	44 (72.1)	20 (41.6)	0.25
31-50	74 (71.2)	31 (46.3)	*0.001*
≥51	68 (66.0)	23 (43.4)	*0.007*
***Pharmacist***	117 (43.3)	54 (35.1)	0.10
18-30	23 (37.7)	17 (51.5)	0.20
31-50	40 (38.5)	20 (29.9)	0.25
≥51	54 (52.4)	17 (32.1)	*0.016*
***Psychologist***	36 (13.3)	6 (3.9)	*0.002*
18-30	10 (16.4)	1 (3.0)	0.05
31-50	13 (12.5)	4 (6.0)	0.16
≥51	12 (11.7)	1 (1.9)	*0.04*
***Exercise-consultant***	145 (53.7)	47 (30.5)	*<0.001*
18-30	39 (63.9)	65 (48.5)	0.15
31-50	68 (65.4)	22 (32.8)	<0.001
≥51	37 (35.9)	9 (17.0)	0.01
***Nurses***	14 (5.2)	18 (11.7)	*0.015*
18-30	3 (4.9)	5 (15.2)	0.09
31-50	5 (4.8)	9 (13.4)	*0.04*
≥51	6 (5.8)	4 (7.5)	0.68
**Program location**			
***Doctor’s clinic***	100 (37.2)	46 (31.3)	0.23
18-30	17 (27.9)	6 (18.8)	0.33
31-50	36 (35.0)	24 (39.3)	0.57
≥51	47 (45.6)	15 (28.3)	*0.04*
***Pharmacy***	*118 (43.7)*	*39 (26.5)*	*0.001*
18-30	22 (36.1)	11 (34.4)	0.87
31-50	46 (44.7)	17 (27.9)	0.03
≥51	50 (48.1)	11 (20.8)	0.001
***Gym***	74 (27.4)	41 (27.9)	0.92
18-30	27 (44.3)	16 (50.0)	0.60
31-50	36 (35.0)	16 (26.2)	0.25
≥51	10 (9.6)	9 (17.0)	0.18
***Workplace***	12 (4.5)	14 (9.5)	*0.04*
18-30	4 (6.6)	2 (6.3)	0.95
31-50	6 (5.9)	9 (14.8)	0.06
≥51	2 (1.9)	3 (5.7)	0.21
***Home***	81 (30.0)	40 (27.2)	0.55
18-30	21 (34.4)	10 (31.3)	0.76
31-50	25 (24.3)	16 (26.2)	0.78
≥51	34 (32.7)	14 (26.4)	0.42
***Community centre***	35 (13.0)	26 (17.7)	0.19
18-30	8 (13.1)	8 (25.0)	0.15
31-50	12 (11.7)	12 (19.7)	0.16
≥51	15 (14.4)	6 (11.3)	0.59
***Other (*** **e.g. *Weight Watchers® clinic)***	12 (4.4)	4 (2.7)	0.38
18-30	1 (1.6)	0	0.47
31-50	2 (1.9)	3 (4.9)	0.25
≥51	9 (8.7)	1 (1.9)	0.10

## Discussion

This study explored the attitudes, perceptions and experiences of women pharmacy consumers in Victoria, Australia and Nottingham, England with currently available weight management products. Overall, similar groups of women in Victoria and Nottingham were surveyed. Approximately 50% of women in Victoria (153/319; 47.9%) and Nottingham (97/188; 51.6%) were classified in the overweight or obese BMI category. The sample is slightly under-representative of the overweight and obese population, which may be explained by the use of self-reported height and weight to calculate the women’s BMI. Studies have previously shown that self-reported height tend to be overestimated and weight is underestimated [28,29].

Women in Nottingham were found to have visited a health care professional in the last 12 months, significantly less than women in Victoria. In Nottingham, close to 50% of women reported visiting their pharmacist for health advice in the last 12 months, significantly less (P < 0.001) than the women in Victoria. In England, it is estimated that 95% of the population visit their community pharmacy once a year [20]. Pharmacy visits are not always related to health and can be for non-medicine purchasers such as toiletries. Our result indicates that visits to the pharmacy may still be associated with prescription drop-off and pick-up rather than a location to receive health care advice. Women who had visited the pharmacist to seek health advice in the last 12 months were significantly more likely to want a pharmacist involved in their ideal weight management program or for their ideal weight management program to be located in a pharmacy. This is in line with previous studies that found people who have had positive experiences with pharmacy services are more likely to feel comfortable approaching pharmacists about health advice [30,31]. Um *et al.*[25] also highlighted that members of the general public were more likely to indicate pharmacies as a potential weight management program location if they had previously received weight management advice by their community pharmacist [25]. Collectively, these results highlight the importance of promoting pharmacists as public health advisors.

Women in Victoria were significantly more likely to be able to demonstrate an understanding of what the benefits of weight loss are in an overweight or obese person. This could be due to the population group in Victoria being significantly more tertiary educated than the women in Nottingham. Decreased blood pressure was the least recognised benefit of weight loss in both women in Victoria and Nottingham. This result is similar to a study conducted in Germany that found obese individuals aged 50–62 were significantly more likely to underestimate their risks of arthritis and hypertension [32]; thus, indicating that people may not understand the importance of weight control on blood pressure and health in general. Pharmacists are in an ideal position to provide information and awareness regarding the benefits of weight loss in an overweight or obese person. Pharmacists should include brief diet and exercise counselling when they first dispense a medication for a condition related to overweight and obesity including commonly dispensed antihypertensive medications. Pharmacists should also reinforce these weight management recommendations with repeat medication dispensing every three to six months. This would encourage a wider understanding of the benefits of weight loss on an overweight or obese person’s health, and for individuals who are in the healthy weight category it would provide a greater understanding of the importance of weight maintenance.

Over 70% of both groups had attempted to lose weight in the past. As in other studies, diet and exercise were commonly used to help achieve weight loss [33-36]. In Victoria, women were significantly more likely to utilise physical activity as part of their weight management strategy compared to women in Nottingham (P < 0.001). Reasons for this finding may include the differences in demographics; women in Victoria were more likely to be educated to a higher level and thus may recognise the importance of including physical activity in a weight management program more readily than the women in Nottingham [37]. Other reasons may include the differences in locations and differences in weather patterns; women in Victoria may be exposed to more parks and outdoor exercise options than women in Nottingham.

Women in Victoria were significantly more likely to utilise pharmacy based programs in the last five years compared to women in Nottingham (P = 0.006). A recent review in Australia revealed 13 different pharmacy weight management programs [38]. The differences between the number of women utilising pharmacy weight management programs in Victoria and Nottingham could be related to the types of pharmacies in which women were surveyed. In Nottingham, women were surveyed only at independently owned pharmacies, whereas in Victoria both privately owned pharmacies and those that are part of a buying group were visited. Women pharmacy consumers surveyed in pharmacies that are part of a group may have utilised pharmacy-based weight management programs more frequently, as many of these programs are run through pharmacy groups and are not available through privately owned pharmacies. Compared to other studies investigating weight management strategies, women in this study were more likely to state that they had utilised herbal products to assist in weight loss in the last five years [33-35]. This could be due to the population sampled. Women pharmacy consumers may be more influenced by the types of products pharmacies sell and thus may be more likely to utilise pharmacy based weight management programs or herbal products compared to the general population.

The weight loss goals, weight loss attainment and duration of the last weight loss attempts differed between the two population groups. Compared to women in Victoria, women in Nottingham were significantly more likely to want to lose more than 10 kgs in their last weight loss attempt (P < 0.001), were significantly more likely to have lost more than 10 kgs (P < 0.001) and were significantly more likely to use their last weight loss method for longer than six months (P = 0.002). Weight loss goal setting has been shown to be a positive influence on achieving weight loss, with national guidelines advising that individuals be encouraged to set realistic weight loss goals (5-10% of their body weight) prior to commencing a weight loss approach [6,8]. Women in Nottingham were also significantly more likely to utilise weight loss support meetings in their weight loss attempt compared to women in Victoria, a strategy that has been shown to achieve more weight loss than diet and/or exercise alone [39,40]. These meetings offer a support network, and provide participants with an environment to learn about different foods, calorie intakes, exercise activities, goal setting, behavioural modification techniques and motivational strategies. Pharmacists and other health care providers can play a role in helping women set realistic weight management goals, offering motivational techniques and strategies to help change behaviour. Pharmacists also see individuals on a regular basis and thus can play a special role in supporting them throughout their weight loss journey. Maher *et al.* found that women viewed pharmacies as a convenient location to receive nutrition advice but highlighted the need to change pharmacy settings, so that they are able to have a more active and supportive role in health prevention [41].

Women in Victoria were significantly more likely to have received advice in their last weight loss attempt from any health care professional (P = 0.001). Nevertheless, the majority of women in both population groups had not received any advice from their health care professional. Primary health care providers are in a special position to offer women weight loss advice. Studies have shown that individuals who have received advice from a health care professional are more likely to be successful in their weight loss approaches [42,43]. Pharmacists, unlike other primary health care providers, also come into contact with both healthy and non-healthy individuals and thus are able to interact with a large population group. Unlike results reported in other studies, women in Nottingham and Victoria felt comfortable receiving advice from their community pharmacist regarding weight management [24, 25_ENREF_25, 30]. The difference may be due to the population group in this study being solely pharmacy consumers, already exposed to the pharmacy environment and likely to be more receptive to receiving advice from their pharmacist. Nevertheless, this shows that pharmacists should feel comfortable offering weight management advice to their consumers. Studies have shown that pharmacists and pharmacy staff may feel uncomfortable providing weight management advice to their consumers due to their lack of training [19,21,22,44]. Increased pharmacy-specific training and education in weight management strategies has been highlighted as an important strategy to improve pharmacy involvement in weight management [19,21].

Overall, women in Victoria and Nottingham had similar ideas on what they wanted in their ideal weight management program. There were however, some reported differences; women in Nottingham were significantly less likely to want a health care professional involved in their ideal weight management program compared to women in Victoria (P = 0.008). Women in Nottingham were less likely to have received advice from a health care professional in their last weight loss attempt compared to women in Victoria (P = 0.001), this may explain why women in Nottingham were more likely to not want any health care professionals in their ideal weight management program. Women in Nottingham may not understand the benefits of having a health care professional as part of their weight management program. In addition, women in Victoria were significantly more likely to want their ideal weight management program to be located in a pharmacy compared to women in Nottingham (P = 0.001). This may be attributed to more women in Victoria having previously tried a pharmacy weight management program in their last weight loss attempt, for this reason women in Victoria may be more familiar or more accepting of weight management programs located in community pharmacies. Although there were some differences the majority of the women in both Victoria and Nottingham wanted advice to be received face-to-face, involve a multi-disciplinary health care team with doctors, dieticians, pharmacists and exercise consultants, and for the program to be available at a convenient location.

Finally, the results from this study have shown that future weight management educational resources developed for women pharmacy consumers in Australia or England have the potential to be used in either country. Furthermore, community pharmacies in countries other than Australia and England may also benefit from pharmacy weight management educational resources to assist pharmacy staff counsel women pharmacy consumers seeking weight management advice. Recently, Richard H. Carmona (17th Surgeon General) in the United States of America, highlighted the importance of pharmacists being involved in chronic disease management and the provision of healthy lifestyle advice [45]. He emphasised that pharmacists are in an ideal position to be health coaches to their consumers [45]. The results from this study have illustrated that women pharmacy consumers in both Victoria and Nottingham are comfortable with pharmacists being involved in the provision of weight management recommendations and have also illustrated the areas in which, women pharmacy consumers need additional advice. Future educational resources for both populations should focus on evidence-based weight management approaches, weight loss goals, weight loss duration and benefits of weight loss. These results may also be used to guide other countries similar to Australia and England to develop their own pharmacy weight management educational resources.

This study only surveyed women pharmacy consumers and thus one of its limitations includes the potential bias towards pharmacy involvement in weight management. A further limitation of this study is that the questionnaires relied on participants remembering information regarding their last weight loss attempt and thus recall bias may be present. The results are, however, similar to other studies that have surveyed the general population regarding previous weight loss experiences [33,46,47].

## Conclusion

This study highlighted the potential for future weight management educational resources developed for women pharmacy consumers in Australia or England to be used interchangeably. Although differences were seen between the two populations, similarities between ideal weight management programs and comfort level with pharmacist interaction were noted. Results from this study have provided information on possible ideal pharmacy weight management programs in both Victoria and Nottingham, which, when developed and implemented, will specifically target the needs of women pharmacy consumers.

## Competing interest

The author(s) declare that they have no competing interests.

## Authors’ contribution

SH was responsible for the design of the study in collaboration with JM, CA and HB. SF designed the questionnaire and was responsible for recruitment, analysis of the data and drafting of the manuscript. SH, JM, CA and HB supervised the data-collection and assisted with data analysis. All authors reviewed and revised the draft manuscript. All the authors read and approved the final manuscript.

## Pre-publication history

The pre-publication history for this paper can be accessed here:

http://www.biomedcentral.com/1471-2458/14/662/prepub

## Supplementary Material

Additional file 1 **Women’s Health and Wellbeing Research Project Questionnaire (Victoria).** Victoria questionnaire. Click here for file

Additional file 2 **Women’s Health and Wellbeing Research Project Questionnaire (Nottingham).** Nottingham questionnaire. Click here for file
