# Supplementary material for: Comparing women pharmacy consumers’ experiences with weight loss treatment in Victoria and Nottingham: a cross-sectional study
Source: BMC Public Health. 2014 Jun 28;14:662. doi: 10.1186/1471-2458-14-662 (PMC4094681; doi:10.1186/1471-2458-14-662)
Supplement: Additional file 1 — Women’s Health and Wellbeing Research Project Questionnaire (Victoria). Victoria questionnaire. [file 1471-2458-14-662-S1.pdf]

MONASH University
Pharmacy and Pharmaceutical Sciences

WOMEN'S HEALTH AND WELLBEING RESEARCH PROJECT QUESTIONNAIRE

Please tick the appropriate box(es) and/or write in the spaces provided.

Section A

1. Why did you visit the pharmacy today? (You may tick more than one)
   - ❑1 To speak to a pharmacist
   - ❑2 To fill a prescription
   - ❑3 To buy an over the counter medication
   - ❑4 To buy a vitamin or herbal product
   - ❑5 To buy cosmetics or perfumes
   - ❑6 To join a weight loss program
   - ❑7 Other (please specify)...

2. How is your health in general?
   ❑1 Very poor ❑2 Poor ❑3 Fair ❑4 Good ❑5 Very good ❑6 Excellent

3. Do you have any medical conditions? (You may tick more than one)
   - ❑1 None
   - ❑2 High cholesterol
   - ❑3 High blood pressure
   - ❑4 Heart condition
   - ❑5 Diabetes
   - ❑6 Cancer
   - ❑7 Depression
   - ❑6 Asthma
   - ❑7 Arthritis
   - ❑8 Others (please specify)..

4. Are you currently taking any medications including non-prescription medications and vitamins (e.g. daily supplements, the oral contraceptive pill, asthma puffers, etc)?
   ❑1 Yes (please specify) ❑2 No
   ....
   ....
   ....

5. Which of the following health care professionals have you visited regarding your health in the last 12 months? (You may tick more than one)
   - ❑1 Doctor
   - ❑2 Pharmacist
   - ❑3 Dentist
   - ❑4 Psychologist
   - ❑5 Physiotherapist
   - ❑6 Dietitian
   - ❑7 Optometrist
   - ❑8 Podiatrist
   - ❑9 Others (please specify)..........................

6. Which single health care professional have you visited **MOST** frequently regarding your health in the last 12 months? (Please tick only one)
   - ❑1 Doctor
   - ❑2 Pharmacist
   - ❑3 Dentist
   - ❑4 Psychologist
   - ❑5 Physiotherapist
   - ❑6 Dietitian
   - ❑7 Optometrist
   - ❑8 Podiatrist
   - ❑9 Others (please specify).........................

7. Do you smoke cigarettes?
   ❑1 Yes *(go to question 10)* ❑2 Never smoked *(go to question 11)* ❑3 I quit *(go to question 8)*

8. How long ago did you quit smoking?
   - ☐1 0-5 months ago ☐2 6-11 months ago ☐3 1-5 years ago
   - ☐4 6-10 years ago ☐5 over 10 years ago

9. Which of the following helped you to quit smoking? (You may tick more than one)
   - ☐1 Quit with no help ☐2 Champix® (Varenicline) ☐3 Zyban® (Bupropion)
   - ☐4 Quit Helpline ☐5 Nicotine replacement therapy *e.g. Patches, gum, lozenges*
   - ☐6 Others (please specify)..

10. Have you ever smoked cigarettes to lose or maintain your weight?
    - ☐1 Yes ☐2 No

11. Do you know how much you currently weigh?
    - ☐1 Yes, please specify: | | | | Kgs **Or** | | | | Pounds
    - ☐2 No

If you would like to know your weight please speak to the research assistant.

12. Do you know how tall you are?
    - ☐1 Yes, please specify: | | | | Cms **Or** | | Feet/inches
    - ☐2 No

If you would like to know your height please speak to the research assistant.

Section B

13. Have **YOU** ever considered yourself overweight?
    - ☐1 Yes ☐2 No

14. Has anyone ever told you that you are overweight?
    - ☐1 Yes *(go to question 15)* ☐2 No *(go to question 16)*

15. Who told you that you were overweight? (You may tick more than one)
    - ☐1 Partner ☐2 Family ☐3 Friends
    - ☐4 Colleagues ☐5 Health care professional (please specify)...

16. What do you believe the benefits of weight loss are? (You may tick more than one)
    - ☐1 No benefits ☐2 Increased energy ☐3 Lower risk of heart problems
    - ☐4 Improved mobility ☐5 Lower risk of diabetes ☐6 Lower risk of high cholesterol
    - ☐7 Decreased blood pressure ☐8 Increased self-esteem ☐9 Increased motivation
    - ☐10 Others (please specify)..

17. Have you **EVER** attempted to lose weight in the past?
    - ☐1 Never ☐2 Once ☐3 2-5 times
    - ☐4 6-10 times ☐5 More than 10 times

If you answered "Never" to question 17 please go to Section C (page 6).
If you ticked another response to question 17 please go to question 18.

18. Why did you want to lose weight? (You may tick more than one)
   - ☐1 To look and feel good ☐2 For a special event ☐3 For my health
   - ☐4 Someone told me to (please specify)...
   - ☐5 Other (please specify)...

19. In the last **FIVE** years which of the following methods have you used to try to lose weight? (You may tick more than one)
   - ☐1 Decreased calorie intake (healthy eating) ☐2 Increased exercise
   - ☐3 Jenny Craig or Weight Watchers (please specify)..
   - ☐4 Meal replacement products, *e.g. Optifast*® (please specify)...
   - ☐5 Pharmacy based weight loss programs, *e.g. Tony Ferguson*® (please specify)..................................
   - ☐6 Weight loss medication, *e.g. Xenical*® (please specify)...
   - ☐7 Vitamins/herbal products marketed for weight loss (please specify)..
   - ☐8 Weight reducing surgery *e.g. gastric banding*
   - ☐9 Others (please specify)...

20. Which of the following methods do you believe are most effective for **SHORT-TERM** weight loss? (You may tick more than one)
   - ☐1 None ☐2 Decreased calorie intake ☐3 Increased exercise
   - ☐4 Jenny Craig or Weight Watchers ☐5 Weight loss medication, *e.g. Xenical*®
   - ☐6 Meal replacement products, *e.g. Optifast*® ☐7 Pharmacy based weight loss programs
   - ☐8 Vitamins/herbal products marketed for weight loss ☐9 Weight reducing surgery
   - ☐10 Others (please specify)...

21. Which of the following methods do you believe are most effective for **LONG-TERM** weight loss? (You may tick more than one)
   - ☐1 None ☐2 Decreased calorie intake ☐3 Increased exercise
   - ☐4 Jenny Craig or Weight Watchers ☐5 Weight loss medication, *e.g. Xenical*®
   - ☐6 Meal replacement products, *e.g. Optifast*® ☐7 Pharmacy based weight loss programs
   - ☐8 Vitamins/herbal products marketed for weight loss ☐9 Weight reducing surgery
   - ☐10 Others (please specify)...

Questions 22-35 are related to your *LAST* weight loss attempt

22. How long ago was your last weight loss attempt? .... years .... months

23. In your ***last*** weight loss attempt, which of the following weight loss methods did you use? (You may tick more than one)
   - ☐1 Decreased calorie intake (healthy eating) ☐2 Increased exercise
   - ☐3 Jenny Craig or Weight Watchers (please specify)..
   - ☐4 Meal replacement products, *e.g. Optifast*® (please specify)...
   - ☐5 Pharmacy based weight loss programs, *e.g. Tony Ferguson*® (please specify)..................................
   - ☐6 Weight loss medication, *e.g. Xenical*® (please specify)...
   - ☐7 Vitamins/herbal products marketed for weight loss (please specify)..
   - ☐8 Weight reducing surgery *e.g. gastric banding*
   - ☐9 Others (please specify)...

24. What influenced you to choose this/these method(s) of weight loss? (You may tick more than one)

❑1 Nothing ❑2 Family/Friends ❑3 TV/radio/newspaper/magazines
❑4 Exercise Consultant e.g. gym instructor ❑5 Internet
❑6 Health care professional (please specify)...
❑7 Other (please specify)...

25. Where was the ***last*** place you purchased a weight loss product (medication/vitamin/herbal) or joined a weight loss program?

❑1 Not applicable ❑2 Supermarket ❑3 Pharmacy
❑4 Internet ❑5 Health food store ❑6 Other (please specify)............................

26. In your ***last*** weight loss attempt how much weight did you **WANT** to lose?

❑1 0-2 kgs ❑2 3-5 kgs ❑3 6-10 kgs
❑4 11-15 kgs ❑5 16-20 kgs ❑6 over 20 kgs

27. How much weight did you lose?

❑1 0-2 kgs ❑2 3-5 kgs ❑3 6-10 kgs
❑4 11-15 kgs ❑5 16-20 kgs ❑6 over 20 kgs

28. How long did you use the weight loss method for?

❑1 0-3 weeks ❑2 1-2 months ❑3 3-5 months
❑4 6-8 months ❑5 9-11 months ❑6 over 1 year

29. Have you since regained any of the weight you lost?

❑1 Yes *(go to question 30)* ❑2 No *(go to question 32)*

30. How long did it take you to regain the weight?

❑1 0-3 weeks ❑2 1-3 months ❑3 4-6 months
❑4 7-11 months ❑5 1-2 years ❑6 over 2 years

31. What do you think caused you to regain the weight? (You may tick more than one)

❑1 Stopped the weight loss method(s) ❑2 Stress
❑3 The weight loss method(s) didn't work ❑4 A significant event
❑5 Other (please specify)...

32. Did you experience any side effects from the weight loss method(s) you ticked in question 23? (You may tick more than one)

❑1 No side effects ❑2 Headache ❑3 Agitation
❑4 Nausea/vomiting ❑5 Constipation ❑6 Loss of concentration
❑7 Diarrhoea ❑8 Other (please specify)...

33. Did you receive advice from a health care professional before you started or while using this/these method(s) of weight loss? (You may tick more than one)

❑1 No advice was received ❑2 Doctor ❑3 Exercise Consultant e.g. gym trainer
❑4 Dietitian ❑5 Pharmacist ❑6 Pharmacy Assistant
❑7 Other (please specify)...

If you answered "No advice was received" to question 33 then please go to question 36.
If you ticked any of the other responses please go to question 34.

34. What advice did the health care professional(s) give you? (You may tick more than one)
❑1 Decrease calorie intake ❑2 Increase exercise ❑3 Take a vitamin/herbal medication
❑4 Take a weight loss medication e.g. *Xenical®, Reductil® or Duromine®*
❑5 Join a weight loss program ❑6 Other (please specify)..

35. Did you find their advice helpful?
❑1 Not at all helpful ❑2 Not helpful ❑3 Unsure
❑4 Somewhat helpful ❑5 Extremely helpful

36. Who/What is your most trusted source for weight loss/maintenance advice?
❑1 Family/Friends ❑2 Internet ❑3 TV/radio/newspaper/magazines
❑4 Exercise consultant ❑5 Health care professional (please specify)...
❑6 No one ❑7 Others (please specify)..

37. How would you feel about a pharmacist giving you advice about weight loss/weight maintenance?
❑1 Not at all comfortable ❑2 Not comfortable ❑3 Unsure
❑4 Somewhat comfortable ❑5 Extremely comfortable

38. What do you think is/are the biggest problem(s) when you are trying to lose or maintain your weight? (You may tick more than one)
❑1 There are no problems ❑2 Lack of motivation
❑3 Lack of support from family and friends ❑4 Lack of time
❑5 Lack of support from health care professionals ❑6 Too little information about what to do
❑7 Side effects of weight loss methods ❑8 Cost of product or program
❑9 Currently available weight loss methods aren't effective
❑10 Other (please specify)..

Questions 39-41 are about your *IDEAL* weight management program.

39. In your program, how would advice and information about weight loss/maintenance be delivered? (You may tick more than one)
❑1 Face to face ❑2 Email ❑3 Telephone calls
❑4 Mobile Phone e.g. SMS ❑5 Postal letter ❑6 Other (please specify)...........................

40. Which health care professional(s) would you like to involve in your program? (You may tick more than one)
❑1 None ❑2 Doctor ❑3 Dietitian
❑4 Pharmacist ❑5 Psychologist ❑6 Exercise Consultant e.g. gym instructor
❑7 Nurse ❑8 Others (please specify)..

41. Where would your program be located?
❑1 Doctors clinic ❑2 Pharmacy ❑3 Gym
❑4 At the workplace ❑5 Home ❑6 Community Centre
❑7 Other (please specify)..

Section C

42. How old are you (in years)?
    - ❑1 18-24 ❑2 25-30 ❑3 31-40
    - ❑4 41-50 ❑5 51-60 ❑6 61-70
    - ❑7 Over 70

43. Are you currently pregnant or breastfeeding?
    - ❑1 Pregnant ❑2 Breastfeeding ❑3 Not pregnant or breastfeeding

44. How many children do you have?
    - ❑1 None ❑2 1 ❑3 2
    - ❑4 3 ❑5 4 ❑6 5
    - ❑6 Other (please specify).....................

45. In which country were you born in?
    ....

46. What is your level of education?
    - ❑1 No formal education ❑2 Primary school or less ❑3 Secondary school or less
    - ❑4 Post secondary school certificate ❑5 University student
    - ❑6 University graduate ❑7 Post graduate

47. What is the postcode of the suburb in which you live?
    ....

THANK YOU FOR YOUR TIME
